# Acetazolamide potentiates the afferent drive to prefrontal cortex in vivo

**DOI:** 10.14814/phy2.13066

**Published:** 2017-01-13

**Authors:** Lezio S. Bueno‐Junior, Rafael N. Ruggiero, Matheus T. Rossignoli, Elaine A. Del Bel, Joao P. Leite, Osvaldo D. Uchitel

**Affiliations:** ^1^ Department of Neuroscience and Behavioral SciencesRibeirao Preto Medical SchoolUniversity of Sao PauloRibeirao PretoBrazil; ^2^ Department of Morphology, Physiology and StomatologyDentistry School of Ribeirao PretoUniversity of Sao PauloRibeirao PretoBrazil; ^3^ Department of Physiology, Molecular and Cell BiologyInstitute of PhysiologyMolecular Biology and NeuroscienceUniversity of Buenos AiresBuenos AiresArgentina

**Keywords:** Carbonic anhydrase, hippocampus, prefrontal cortex, single‐unit activity, synaptic plasticity

## Abstract

The knowledge on real‐time neurophysiological effects of acetazolamide is still far behind the wide clinical use of this drug. Acetazolamide – a carbonic anhydrase inhibitor – has been shown to affect the neuromuscular transmission, implying a pH‐mediated influence on the central synaptic transmission. To start filling such a gap, we chose a central substrate: hippocampal‐prefrontal cortical projections; and a synaptic phenomenon: paired‐pulse facilitation (a form of synaptic plasticity) to probe this drug's effects on interareal brain communication in chronically implanted rats. We observed that systemic acetazolamide potentiates the hippocampal‐prefrontal paired‐pulse facilitation. In addition to this field electrophysiology data, we found that acetazolamide exerts a net inhibitory effect on prefrontal cortical single‐unit firing. We propose that systemic acetazolamide reduces the basal neuronal activity of the prefrontal cortex, whereas increasing the afferent drive it receives from the hippocampus. In addition to being relevant to the clinical and side effects of acetazolamide, these results suggest that exogenous pH regulation can have diverse impacts on afferent signaling across the neocortex.

## Introduction

A brain area can manifest a field postsynaptic potential (fPSP) in response to an afferent electrical pulse. Delivering a second pulse within dozens of milliseconds reevoke that fPSP, but usually at decreased or enhanced voltage amplitude. The latter is referred to as paired‐pulse facilitation (PPF): a form of experimentally induced synaptic plasticity present in the neocortex and hippocampus (Zucker and Regehr 2002). It is believed to mimic endogenous rapid input changes, which could partially underlie sensory integration, working memory, and their dysfunctions (Bartlett and Smith 2002; Skaliora et al. 2004; Lee et al. 2007; Jentsch et al. 2009; Kiss et al. 2011). PPF is considered to have a presynaptic nature, as it would reflect a transiently higher probability of neurotransmitter release due to a boost in vesicle exocytosis (Zucker and Regehr 2002). Such a boost is known to depend on the residual cytosolic concentration of presynaptic Ca^2+^ ions caused by trains of action potentials (Katz and Miledi 1968), and the facilitation of presynaptic Ca^2+^ currents through voltage‐gated channels (Inchauspe et al. 2004). Both would potentiate vesicle docking and quantal release (Wang et al. 1996; Moresco et al. 2003; Srinivasan et al. 2008).

Experimentally elevating or buffering presynaptic Ca^2+^, respectively, reinforces or undermines PPF (Zucker and Regehr 2002). An alternative experimentation without directly manipulating Ca^2+^ is to inhibit the carbonic anhydrase, which regulates pH through catalyzing the carbonic acid and carbon dioxide plus water interconversion. Uchitel and Groisman (2014) have shown that the carbonic anhydrase inhibitor acetazolamide (AZ) reduces the amplitude of end‐plate potentials in ex vivo neuromuscular junctions. The authors have also revealed a >60% retention of fluorescent‐dyed presynaptic vesicles under AZ, implying that this drug could affect PPF in the brain. AZ has long‐accepted medical uses against epileptic seizures (Ansell and Clarke 1956; Millichap 1987; Reiss and Oles 1996; Hamidi and Avoli 2015), as well as side effects on cognition (Sun et al. 2001; Wang et al. 2013; Yang et al. 2013). Thus, both properties could partially result from central PPF alterations, which remain unexplored.

There is a source of hippocampal formation outputs, the intermediate CA1/subiculum area (CA1/sub), whose communication with the rat medial prefrontal cortex (mPFC) is prone to PPF (Izaki et al. 2002, 2003; Takita et al. 2013), and is implicated in epilepsy and cognition (Meyer‐Lindenberg et al. 2005; Szyndler et al. 2009; Ma and Leung 2010; Kleen et al. 2011; Holmes et al. 2015; McGarrity et al. 2016). This pathway could, therefore, participate in systemic AZ effects, which we evaluated through PPF and single‐unit recording in behaving rats.

## Materials and Methods

## Subjects

Pairs of adult male Wistar rats (350–400 g; N = 10) were housed in standard bedded cages with food and water ad libitum. The colony room was maintained at 24°C and 12 h light/dark cycle (lights on at 7 am). Procedures followed the Brazilian Council for the Control of Animal Experimentation guidelines, and were approved by the Ribeirao Preto Medical School bioethics committee (128/2014).

### Chronic implants

Rats were anesthetized with ketamine (0.75 mg/Kg intraperitoneal, ip, then 0.5 mg/Kg intramuscular, im) and xylazine (0.38 mg/Kg ip, then 0.25 mg/Kg im) hydrochlorides. Stereotaxic and craniotomy procedures aimed at two ipsilateral coordinates in the left hemisphere. Prelimbic area of the mPFC: 3.1 mm anterior to bregma, 0.5 mm lateral to midline, and 3.1 mm ventral to dura. CA1/sub of the intermediate hippocampal formation: 5.7 mm posterior to bregma, 4.5 mm lateral to midline, and ~2.5 mm ventral to dura (Paxinos and Watson 2007). A recording microelectrode bundle consisting of 16 microwires (teflon‐coated tungsten, 50 *μ*m bare diameter, California Fine Wires) soldered to a pair of eight‐channel Omnetics connectors was implanted in the mPFC. For electrical stimulation of the CA1/sub area we implanted a twisted bipolar electrode (teflon‐coated tungsten, 60 *μ*m bare diameter, ~500 *μ*m tip separation, AM‐Systems). Six microscrews were fastened on skull around those implants, one of which serving as ground, touching the contralateral cerebellum. Microscrews and electrodes were enclosed together with acrylic cement, and the rats were finally treated with flunixin meglumine (analgesic, anti‐inflammatory, and antipyretic) and veterinary antibiotics. After surgery, rats were individually housed for a 5‐ to 7‐day recovery period.

### Experimental design

Rats were handled for habituation ~24 h before the experiment. On the experiment day, rats were plugged to a stimulating/recording cable. They were then placed in a standard operant chamber – located in a soundproof wooden box – without behavioral contingencies, sensory stimuli, food/water troughs, or levers/nose‐poke apertures. Once connected to the electrophysiological equipment, the stimulating/recording cable became suspended from above this chamber, thus allowing the rats to move freely.

**Figure 1:**
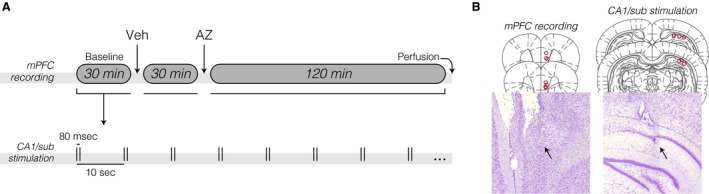
Experimental design and electrode placements. (A) Chronic electrophysiological timeline, undertaken once per rat after a 5‐ to 7‐day post‐surgical recovery. Vehicle (Veh) and acetazolamide (AZ) solutions were administered via the intraperitoneal route while keeping the rat plugged into the electrophysiological cable. The medial prefrontal cortex (mPFC) was recorded while stimulating the intermediate hippocampal formation CA1/subiculum area (CA1/sub) throughout the session. (B) Top: coronal sections (Paxinos and Watson 2007) with electrolytic lesions across rats indicated by red circles. Bottom: Nissl‐stained specimens from two representative subjects. The arrows point to electrolytic lesions.

A single group was used, that is, rats were their own controls. The experiment consisted of a single session divided into: 30 min baseline recording – 5 min pause for vehicle injection (ip) – 30 min postvehicle recording – 5 min pause for AZ injection (ip) – 120 min post‐AZ recording (Fig. 1A), throughout which the CA1/sub was stimulated and the mPFC was recorded. A 15 min habituation preceded the experiment, and the rats were kept plugged to the cable at all times to ensure consistency of electrophysiological signals.

After the experiment, rats were anesthetized, perfused, and decapitated. Before skull opening, electrode tips were marked with electrolytic lesions (0.8 mA, 0.8 sec monophasic current). Brains were then removed from the cranial vault, postfixed in 4% paraformaldehyde (4 h), and cryoprotected in 20% sucrose overnight. Upon cryostat 30 *μ*m sectioning, specimens were mounted on glass slides for Nissl staining, bright‐field microscopy, and electrode positioning validation (Fig. 1B).

### Drug dose and vehicle

The lipophilic AZ powder was diluted to 400 mmol/L in pure DMSO. AZ + DMSO was further diluted to 50% in saline, and this solution was delivered via the ip route. The AZ dose was 40 mg/Kg, resulting in injected volumes no greater than 0.4 mL per rat. The control vehicle (Veh) was 50% DMSO in saline.

### Electrophysiology

A Grass Technologies S88X stimulator was used to generate electrical pulses, which were photoelectrically isolated through a PSIU6X unit before reaching the brain. Monophasic square pulses (200 *μ*sec, 200–300 *μ*A, i.e., ~70% of input–output curve; Bueno‐Junior et al. 2012; Lopes‐Aguiar et al. 2013) were delivered in pairs (80 msec interpulse interval) into the CA1/sub at 0.1 Hz. We chose the 80 msec interpulse interval for optimal PPF based on the literature (Laroche et al. 1990; Izaki et al. 2003; Kiss et al. 2011); using a single interpulse interval, rather than a series of them, allowed us to standardize fPSP responses, which could then be analyzed as repeated measures (see Fig. 1A and the Data analysis subsection). Paired‐pulse delivery at 0.1 Hz was also based on previous reports (Goto and O'Donnell 2002; Kiss et al. 2011); probing PPF at 0.1 Hz, rather than lower frequencies (e.g., 0.05 Hz), allowed us to increase the fPSP sampling, and therefore the data yield.

A Plexon Multichannel Acquisition Processor (MAP, 16 channels) was employed to record mPFC field potentials and multiunit activity. Parameters were as follows. Field potentials: 0.7–500 Hz band‐pass filter, 1000× gain (PBX preamplifier), and 2 kHz digitization (computer). Multi‐unit activity: 250–8000 Hz band‐pass filter, 1000x gain (PBX), and 40 kHz digitization (MAP). For each CA1/sub paired pulse, a corresponding digital event from the S88X stimulator was input into the MAP at 40 kHz digitization. We used principal component analysis, and cluster delimitation to remove CA1/sub pulse artifacts before moving on to semiautomatic spike sorting (Offline Sorter, Plexon). Sorted clusters were further qualified by a signal/noise ratio criterion (>2), and then accepted as single‐unit activity data.

### Data analysis

**Figure 2:**
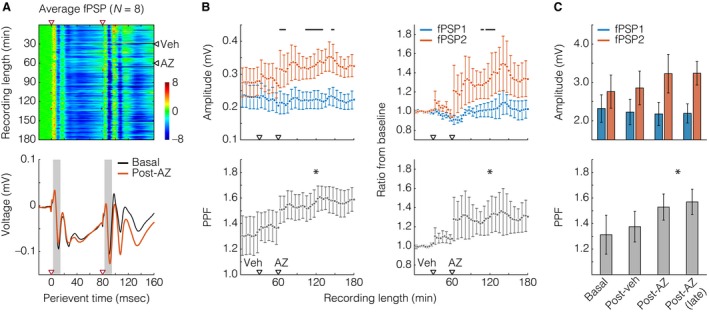
Acetazolamide (AZ) enhanced the hippocampal‐prefrontal cortical paired‐pulse facilitation (PPF). (A) Top: heat plot with field postsynaptic potential (fPSP) sweeps along dimension 1 (Y) and perievent time in dimension 2 (X). Paired‐pulse electrical stimuli were delivered once every 10 s, for a total of 1080 sweeps (180 basal, 180 post‐vehicle, Veh, 720 post‐AZ). fPSP voltage values were Z‐scored (color bar) against the 20 msec prepulse period. Red and black triangles situate paired‐pulse stimuli and injections, respectively. Heat‐plot data are from averaging across rats (*N* = 8). Bottom: average basal and post‐AZ fPSP voltage traces from the heat‐plot data. Gray areas illustrate the time windows from which amplitudes were measured. (B) Top left: fPSP1 (blue) versus fPSP2 (orange) amplitudes. Each time point represents 18 fPSP, that is, 3 min. Black lines indicate Tukey's post hoc differences after two‐way repeated measures ANOVA. Bottom left: PPF, that is, ratios between fPSP2 and fPSP1. The asterisk informs an effect of time, after one‐way repeated measures ANOVA. Right: same data, but shown as ratios from baseline means. (C) Data from the left graphs of panel B, but averaged across four periods: baseline, post‐Veh, initial 30 min post‐AZ, and final 30 min post‐AZ. The asterisk in the inferior graph informs an effect of time, after one‐way repeated measures ANOVA. All data are shown as mean ± standard error.

**Figure 3:**
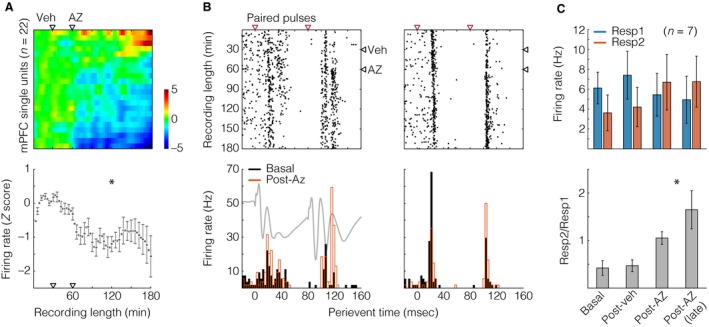
Acetazolamide (AZ) effects on medial prefrontal cortical (mPFC) firing combined net inhibition with increased paired‐pulse responsivity in a minor portion of neurons. (A) Top: heat plot with single units (i.e., their nonperievent rate histograms) along dimension 1 (Y) and time bins (3 min width) in dimension 2 (X). Spike counts were Z‐scored (color bar) against the initial 15 min baseline period. Black triangles represent injections. Bottom: Mean ± standard error from the heat‐plot data (*n* = 22). The asterisk indicates an effect of time, after one‐way repeated measures ANOVA. (B) Two representative neurons: their perievent raster plots (top), and correspondent 3 msec‐binned histograms (bottom). Red triangles situate paired‐pulse stimuli, and post‐AZ firing rates are from the initial 30 min post‐AZ period. A subjacent voltage trace from Figure 2 (left histogram) illustrates the timing between field and unit responses to hippocampal stimuli. (C) Top: firing responses to conditioning (Resp1) and test pulses (Resp2) after counting spikes within 15–42 poststimulus latencies. Bottom: ratios between Resp2 and Resp1 firing rates. The asterisk informs an effect of time, after one‐way repeated measures ANOVA. Data were averaged across four periods: baseline, post‐Veh, initial 30 min post‐AZ, and final 30 min post‐AZ. All data are shown as mean ± standard error.

Using custom Matlab codes, 180‐msec perievent windows comprising fPSP (20 msec prior to conditioning pulse, 80 msec after conditioning pulse, and 80 msec after test pulse) were extracted from the field potential data. We could then automatize amplitude measurements within 5–14 msec postpulse latencies (Fig. 2A), and average them every 3 min (i.e., 18 fPSP), or into four 30 min periods: baseline, post‐Veh, initial 30 min post‐AZ, and final 30 min post‐AZ. Two analyses were made thereafter: (1) fPSP1 and fPSP2 amplitudes, and their ratios from the mean amplitude of the initial 15 min baseline; and (2) PPF values, that is, fPSP2 divided by fPSP1 amplitudes, and their ratios from baseline. Single‐unit activity, in turn, was analyzed as: (1) nonperievent spike count histograms (3 min bin width) Z‐scored against the initial 15 min baseline; and (2) perievent firing rate histograms (180 msec perievent windows as above, 3 msec bin width). Firing responses to either conditioning (Resp1) or test pulses (Resp2) were counted within 15–42 msec postpulse latencies (Fig. 3B), and averaged into four 30 min periods as above. Values were then plotted as either spikes per second or Resp2/Resp1 ratios. Statistical evaluations were made through repeated measures ANOVA at the *P* ≤ 0.05 significance criterion: either one‐way (when analyzing PPF, Resp2/Resp1 ratio, and nonperievent neural activity) or two‐way (when comparing fPSP1 vs. fPSP2, and Resp1 vs. Resp2).

## Results

Two of the 10 subjects were excluded from analysis, as they did not present consistent fPSP responding. Figure 2A describes the average prefrontal fPSP from the eight analyzed rats. The most consistent fPSP waveform we found here was a W‐shaped voltage deflection, with the first and second valleys at the 10–12 msec and 26–28 msec poststimulus latencies, respectively. The 10–12 msec latency presumptively reflects monosynaptically driven depolarizations (Ferino et al. 1987; Parent et al. 2010; Sloan et al. 2011), and was therefore chosen for the amplitude measurements of Figure 2B–C. The left graphs of Figure 2B show that AZ enhanced the amplitude of fPSP2, but not fPSP1 (near significant between fPSP effect: *F*_(1,826) _= 3.716, *P* = 0.074, power of test = 0.323; significant effect of time: *F*_(59,826) _= 1.355, *P* = 0.043, power of test = 0.507; significant fPSP vs. time interaction: *F*_(59,826) _= 1.613, *P* = 0.003, power of test = 0.836). The fPSP2/fPSP1 ratio, that is, PPF, was also increased by AZ (significant effect of time: *F*_(59,413) _= 4.161, *P* < 0.001, power of test = 1). The right graphs of Figure 2B are from the same data, but shown as the ratio from baseline: more specifically, the ratio from the mean amplitude of the initial 15 min baseline period. This calculation resulted in no clear distinctions between the two halves of the baseline period, making sure the statistically insignificant post‐Veh deviation from baseline is not artifactual. We again found that AZ potentiated fPSP2 responses (significant effect of time: *F*_(59,826) _= 2.580, *P* < 0.001, power of test = 1; near significant fPSP vs. time interaction: *F*_(59,826)_ = 1.331, *P* = 0.053, power of test = 0.468), and hence the PPF (significant effect of time: *F*_(59,413) _= 2.312, *P* < 0.001, power of test = 0.999). The mean ± standard error bars of Figure 2C are also from the left graphs of Figure 2B, but collapsed into four 30 min periods. No statistical effect was found when comparing raw fPSP amplitudes, but we again detected a significant PPF increase under AZ (significant effect of time: *F*_(3,21) _= 4.233, *P* = 0.017, power of test = 0.656). Thus, fPSP amplitude measurements demonstrate that systemic AZ had a PPF‐potentiating effect in the hippocampal‐prefrontal circuit.

Our spike recordings yielded 22 mPFC single units across eight rats, none identified as putative fast‐spiking interneurons, according to criteria of firing rate and action potential width (Homayoun and Moghaddam 2007). Therefore, our sample of units is supposedly formed by principal excitatory neurons. The top image of Figure 3A represents a 2D array of spike count histograms, with units along dimension 1 and time bins (3 min) in dimension 2. Similarly to Figure 2 analyses, *Z* scores are based on the initial 15 min baseline. Histograms were sorted from top to bottom according to the mean *Z* score (the lower the value, the lower the row), and the array was plotted as image with scaled colors. This image is an overview of mPFC activity before and after ip injections, demonstrating that: (1) there were no clear changes throughout the baseline and post‐Veh period, as expected; and (2) most of units had their activity reduced by AZ. The mean ± standard error curve below the image confirms such a reduction (effect of time: F_(59,1239) _= 5.601, *P* < 0.001, power of test = 1), which is actually a net inhibition, because a minority of units had a late‐onset increase in firing (Fig. 3A). Seven of 22 mPFC units manifested consistent responses to CA1/sub stimuli. Two of them are depicted in Figure 3B as perievent raster plots (top) with correspondent 3 msec‐binned rate histograms (bottom), comparing the baseline versus the initial 30 min after AZ. A subjacent voltage trace illustrates the timing between fPSP and unit responses. In general, Figure 3B suggests that AZ potentiated responses to test pulses (Resp2). This is reinforced by Figure 3C, showing Resp1 versus Resp2 raw values and Resp2/Resp1 ratios throughout the recording period. The ANOVA returned a significant response versus time interaction when comparing Resp1 and Resp2 (*F*_(3,36) _= 4.286, *P* = 0.011, power of test = 0.704). Moreover, consistently with the PPF results, there was a significant increase in the Resp2/Resp1 ratio (*F*_(3,18) _= 8.313, *P* = 0.001, power of test = 0.961). This ratio increase probably derives from the subtly opposite reactions of Resp1 and Resp2 to AZ, which could motivate new studies with larger samples of units. Altogether, these findings indicate that AZ inhibited the overall mPFC firing, but potentiated hippocampus‐elicited responses in a minor portion of its recorded neurons.

## Discussion

This study provides short‐term plasticity and single‐neuron data on the central AZ effects in vivo. While reducing neocortical firing, AZ strengthened hippocampus‐induced presynaptic plasticity, suggesting a shift toward afferent drive. Although this seems true for projections between CA1/sub and mPFC, other axonal pathways could behave differently under AZ. In fact, Uchitel and Groisman (2014) have shown an opposite effect in the neuromuscular transmission. Also, Takita et al. (2013) have suggested that the factors underlying hippocampal‐prefrontal cortical PPF – such as presynaptic Ca^2+^ concentration and feedforward interneuronal processing – depend on which hippocampal region is being stimulated, either intermediate or ventral. Therefore, the probably diverse effects of systemic AZ throughout the nervous system are underexplored, in contrast to the wide clinical use of this drug (e.g., Reiss and Oles 1996; Kaur et al. 2002; Vagal et al. 2009; Heming et al. 2012; Ritchie et al. 2012; Kotagal 2012; Supuran 2015).

Systemic AZ crosses the blood–brain barrier (Hanson et al. 1981; Collier et al. 2016). Once in the brain, AZ inhibits the carbonic anhydrase, thus diminishing the buffering capacity (Heuser et al. 1975). As reviewed by Chesler (2003), physiological and disease conditions also modulate proton concentration in the brain. Increase in proton concentration or reduction in pH activates or inhibits specific channels, like acid‐sensing ion channels or calcium channels, in addition to modulating ligand‐gated ion channels such as NMDA and GABA receptors. It is generally considered that acidification reduces and alkalinization increases neuronal excitability (Chesler 2003). Furthermore, slight fluctuations in intracellular or extracellular pH can affect protein function, cellular metabolism, and the electrical machinery of neuronal and glial cells (Zeng and Xu 2012). At the synaptic level, key components of vesicular neurotransmitter release are affected by cytosolic pH, which, for example, underlie the endocytosis‐inhibiting effects of acidification (Dejonghe et al. 2016), suggesting that the prolonged poststimulation alkalinization facilitates endocytosis (Zhang et al. 2010). This pH shift is greatly enhanced by inhibiting with AZ the action of carbonic anhydrase. Therefore, AZ may change the mode of vesicle recycling, thereby affecting PPF via modulation of the number of docked vesicles.

The CA1/sub‐mPFC pathway is critically involved in working memory (Takita et al. 2013), as are short‐term synaptic processes recruited by PPF within the hippocampus (Silva et al. 1996). Disrupted PPF in the CA1/sub‐mPFC pathway itself has been implicated in a pharmacological model of psychosis (systemic NMDA antagonism), which moreover was shown to reduce mPFC neural activity (Kiss et al. 2011). These findings are similar to those reported here, suggesting that our data might be related to the aforementioned influence of pH on ionotropic receptors. Altered hippocampal‐prefrontal function has been associated with cognitive deficits and schizophrenia (Meyer‐Lindenberg et al. 2005; McGarrity et al. 2016), consistently to a detrimental role of AZ on human working memory (Wang et al. 2013) and rodent learning (Sun et al. 2001; Yang et al. 2013). Given the myriad of AZ clinical uses and behavioral side effects, more in vivo experiments like ours are required for elucidating how carbonic anhydrase impacts interareal brain communication. Promising approaches include the assessment of AZ effects on different PPF protocols (Zucker and Regehr 2002) or long‐term forms of synaptic plasticity (Citri and Malenka 2008) in combination with invasive pH monitoring of specific brain sites (Zhou et al. 2016). Mechanistic explorations should also study selective intracellular versus extracellular carbonic anhydrase inhibitors (e.g., Perez Velazquez 2003; Makani et al. 2012; Collier et al. 2016) in their effects on presynaptic plasticity.

## Conflict of Interest

No conflicts of interest, financial or otherwise, are declared by the authors.
